# Molecular mapping of a new recessive wheat leaf rust resistance gene originating from *Triticum spelta*

**DOI:** 10.1038/s41598-020-78679-3

**Published:** 2020-12-17

**Authors:** Vishal Dinkar, S. K. Jha, Niharika Mallick, M. Niranjana, Priyanka Agarwal, J. B. Sharma, Vinod

**Affiliations:** Division of Genetics, ICAR-Indian Agricultural Research Institute, New Delhi, 110012 India

**Keywords:** Genetics, Plant sciences

## Abstract

TSD276-2, a wheat genetic stock derived from the cross Agra Local/*T. spelta* 276 showed broad spectrum resistance against leaf rust pathogen. Genetic analysis was undertaken using F_1_, F_2_, F_2:3_ and BC_1_F_1_ generations derived from the cross TSD276-2/Agra Local. The results revealed a single recessive gene for leaf rust resistance, tentatively named as *LrTs*_*276-2*,_ in TSD276-2. Molecular mapping of leaf rust resistance gene *LrTs*_*276-2*_ in TSD276-2 was done using SNP-based PCR and SSR markers. For Bulked Segregant Analysis (BSA), two bulks viz. resistant bulk and susceptible bulk, and the parents TSD276-2 and Agra Local were genotyped for SNPs using AFFYMETRIX 35K Wheat Breeders' AXIOM array. *T. spelta* 276 was also genotyped and used as a check*.* BSA indicated that the gene for leaf rust resistance in TSD276-2 is located on chromosome arm 1DS. Putatively linked SNPs on chromosome arm 1DS were converted into PCR-based markers. Polymorphic SSR markers on chromosome arm 1DS were also identified. Final linkage map was constructed using one SNP-based PCR and three SSR markers. The rust reaction and chromosomal location suggest that *LrTs*_*276-2*_ is a new leaf rust resistance gene which may be useful in broadening the genetic base of leaf rust resistance in wheat.

## Introduction

Leaf rust caused by *Puccinia triticina* Eriks. is one of the most important and widespread foliar diseases of wheat (*Triticum aestivum* L.) inflicting significant yield losses in susceptible cultivars^1–4^. Although, rust diseases can be controlled by application of fungicides, genetic resistance remains the most effective, economical and environmentally sustainable method^5,6^. To date, 79 leaf rust resistance genes have been designated and catalogued in wheat and about half of them have their origin in various closely or distantly related species and genera of wheat while remaining resistance genes are native to wheat^7–9^. Many of the leaf rust resistance have become ineffective due to evolution of new virulent pathotypes. This necessitates continuous search for new and effective resistance genes for deployment in wheat cultivars. Spelt wheat (*T. spelta*) is potentially a good source of rust resistance genes^10^. *T. spelta* is a hulled wheat and is considered as ancestral to the free-threshing forms of hexaploid wheat^11^. Spelt wheat shares the same genomic structure, 2*n* = 6*x* = 42 (BBAADD genome) with common wheat and belongs to primary gene pool of wheat. This facilitates gene transfer from *T. spelta* by direct hybridization through homologous recombination. Till date, only three leaf rust resistance genes viz., *Lr44* on chromosome 1B^10^, *Lr65* on chromosome 2A^12^ and *Lr71* on chromosome 1B^13^ from spelt wheat have been identified and mapped.

As part of our pre-breeding programme at Indian Agricultural Research Institute, New Delhi, we have been working to identify and map rust resistance genes from primary, secondary and tertiary gene pools^14–19^. The present study reports the inheritance and molecular mapping of leaf rust resistance in *T. spelta* derived bread wheat line TSD276-2.

## Material and methods

### Plant materials

*Triticum spelta* derived bread wheat line TSD276-2 and leaf rust susceptible cultivar Agra Local (AL) were used to study the mode of inheritance and molecular mapping of leaf rust resistance. TSD276-2 is derived from the cross *T. spelta* accession 276/Agra Local. *T. spelta* accession 276 (*T. spelta*276) is a winter wheat requiring either vernalization or a prolonged photoperiod for flowering while AL is spring wheat. TSD276-2 showed spring wheat nature with no vernalization requirement. The F_1_, F_2_ and F_2:3_ population from the cross TSD276-2/AL were used for genetic analysis and molecular mapping of leaf rust resistance. *T. spelta* 276, the donor of leaf rust resistance to TSD276-2 was also used as a check in this study.

### Leaf rust pathotypes

Pure inoculum of *Puccinia triticina* pathotypes was obtained from ICAR-Indian Institute of Wheat and Barley Research, Regional Station, Flowerdale, Shimla. Pathotypes were multiplied and maintained on susceptible cultivar AL under greenhouse conditions at Division of Genetics, IARI, New Delhi. *T. spelta* 276 and its derivative TSD276*-*2 along with susceptible check Agra Local were tested with 17 diverse *Puccinia triticina* pathotypes during crop season 2017–2018 (Table 1). Pathotype 77-5 (121R63-1), currently one of the most predominant one in India, was used for genetic analysis and molecular mapping.

**Table 1:** Infection types on Agra Local, *T. spelta* 276 and TSD276-2 against 17 pathotypes of *P. triticina* when tested at seedling stage at mean temperature range of 20–28 °C.

S. no	Pathotypes of *P. triticina*	Agra local	*T. spelta* 276	TSD276-2
(1)	77	3^+^	;^N^	;
(2)	77A	3^+^	0;	0;
(3)	77A-1	33^+^	;1	0;
(4)	77-2	33^+^	;	;
(4)	77-3	33^+^	;	0;
(5)	77-4	33^+^	;^N^	;
(6)	77-5	33^+^	;1^=N^	;1^−N^
(7)	77-6	33^+^	;^N^	;1^−N^
(8)	77-8	3	0;	0;
(9)	77-9	3	;	;1^−^
(10)	77-10	33^+^	;	;
(11)	104	3^+^	;	0;
(12)	104-4	33^+^	;^N^	;
(13)	106	3^+^	0;	0;
(14)	108	3^+^	;	;1^−^
(15)	162A	3^+^	;1^=^	0;
(16)	12-3	33	;	;
(17)	12-4	3^+^	;	;

### Screening for leaf rust resistance

Screening for leaf rust resistance was done at seedling stage in greenhouse. Seeds were sown in small rectangular metallic trays (28 cm × 10 cm × 7.5 cm). About 10 day old seedlings were inoculated by spraying an aqueous suspension of *P. triticina* uredospores. The uredospore suspension was mixed with a drop of Tween20. Inoculated seedlings were incubated in a humid glass chamber for 48 h and were subsequently transferred to benches in a greenhouse under ambient condition of light and relative humidity. Disease reaction was recorded 12 days after inoculation as per the method described by Stakman et al.^20^.

### Molecular marker analysis

Fresh leaf samples collected from 40 to 45 days old plants were crushed in liquid nitrogen with mortar and pestle. DNA isolation was done following CTAB method^21^. DNA was quantified on 0.8% (w/v) agarose gel using Lambda Uncut DNA (THERMO FISHER SCIENTIFIC INC., USA) as standard and confirmed with NanoDrop Lite spectrophotometer (THERMO FISHER SCIENTIFIC INC., USA). DNA was diluted to the working stock concentration of 25 ng/μL and stored at − 20 °C.

For bulked segregant analysis equal amount of DNA from 20 homozygous resistant (HR) and 20 homozygous susceptible (HS) lines from F_2:3_ population was bulked to constitute two contrasting bulks viz*.* resistant bulk and susceptible bulk^22^. *T. spelta*276, TSD276-2, Agra Local and two extreme bulks i.e. RB and SB were genotyped for SNP using AFFYMETRIX 35K Wheat Breeders' AXIOM array^23^. SNPs found to be polymorphic between parents as well as bulks were identified. Chromosomal region found to show maximum polymorphic SNPs between bulks was presumed to carry leaf rust resistance gene. These SNPs were converted to PCR based markers using the software primer 3(v.0.4.0) as described earlier^19^. These SNP-based PCR markers were used for parental polymorphism.

Besides, a total 51 SSR markers spanning across the putatively identified chromosome 1D (on the basis of SNP genotyping) carrying rust resistance gene were tested for polymorphism between TSD276-2 and AL^24,25^. Primer sequences of these markers are available in public domain (https://wheat.pw.usda.gov/cgi-bin/GG3/browse.cgi?class=marker). For studying marker polymorphism between parents, PCR was performed in a reaction volume of 10 μl. Each 10 μl reaction volume included 2 μL of template DNA (50 ng), 1 μL forward primer (5 pm/μl), 1 μL of reverse primer (5 pm/μl), and 3 μL of Taq DNA Polymerase RED 2× master mix (AMPLIQON A/S, Denmark) and 3 μL of nuclease free water (THERMO FISHER SCIENTIFIC INC., USA). The PCR reactions were performed in 96-well PCR plates with thermal seal in an APPLIED BIOSYSTEMS VERITI thermal cycler at specific profile. The PCR conditions for primers used are given in Table 2. PCR amplified products were resolved on 3.5% (w/v) Agarose (LONZA, Rockland, USA) gel stained with ethidium bromide. Gels were visualized with a UV-transilluminator gel documentation system (SYNGENE G-BOX, Cambridge, UK).

**Table 2:** PCR amplification conditions of molecular markers used in genetic map construction.

Marker	Initial denaturation	Denaturation	Annealing	Extension	Total cycles	Final extension
SNP AX-94393474	94 °C for 4 min	94 °C for 30 s	60 °C for 30 s	72 °C for 30 s	35	72 °C for 10 min
SSR *Xcfd15*	94 °C for 4 min	94 °C for 30 s	60 °C for 30 s	72 °C for 30 s	35	72 °C for 10 min
SSR *Xcfd61*	94 °C for 4 min	94 °C for 30 s	60 °C for 30 s	72 °C for 20 s	30	72 °C for 10 min
SSR X*gwm106*	94 °C for 4 min	94 °C for 30 s	60 °C for 30 s	72 °C for 20 s	30	72 °C for 10 min

Finally, both polymorphic SNP-based PCR markers and polymorphic SSR markers were used for bulked segregant analysis to confirm the identity of chromosome carrying leaf rust resistance gene^22^. Total 136 F_2:3_ homozygous lines were genotyped with SNP-based PCR marker as well as SSR markers identified as polymorphic in BSA. Linkage analysis was performed using MAPMAKER v3.0^26^ with a minimum LOD score of 3.0 and a maximum genetic distance of 37.2 cM. ‘COMPARE’, ‘TRY’ and ‘RIPPLE’ commands of MAPMAKER v3.0 were used to check the final order of map. The genetic distances (cM) were calculated using the Kosambi mapping function^27^. Chi-square test was conducted to test the goodness-of-fit for segregation of the resistance gene^28^. Putative gene(s) present between flanking marker interval were predicted using wheat sequence (International Wheat Genome Sequencing Consortium, 2018) available at Ensembl Plants (https://plants.ensembl.org/Triticumaestivum/Info/Index) between the two flanking markers utilizing BioMart (https://plants.ensembl.org/biomart/martview/12b2b93c60bfbcedcaf0e4d1e023fee9).

## Results

### Genetic analysis of leaf rust resistance

**Figure 1:**
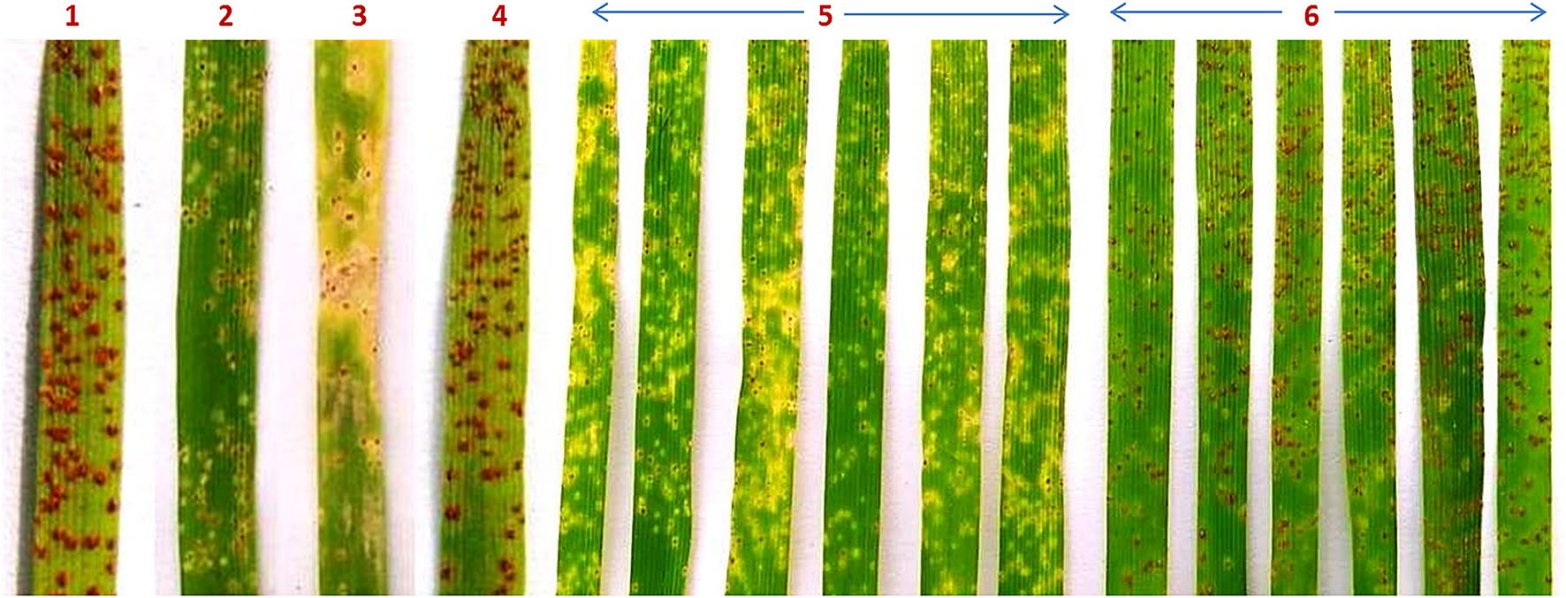
Infection types (ITs) of pathotype 77-5 on (1) Agra Local, (2) *T.spelta* 276, (3) TSD276-2, (4) F_1_ (TSD276-2/AL), (5) HR F_3_, and (6) HS F_3_ .

TSD276-2 showed high degree of leaf rust resistance with ITs ranging from “0;” to “1^−”^ against different *P. triticina* pathotypes, whereas the susceptible parent Agra Local showed susceptible reaction with infection type (IT) “3” to “33^+”^ against all the pathotypes used in the study (Fig. 1). The original spelt wheat accession *T. spelta* 276 also showed high degree of resistance against all the 17 pathotypes (Table 1).

For genetic analysis, TSD276-2, AL, F_1_ (TSD276-2/AL) and 294 F_2_ plants were screened for leaf rust resistance against *P. triticina* pathotype 77-5. TSD276-2 showed resistance reaction with ITs “;1^−N^” whereas Agra Local showed susceptibility (IT 33^+^). All the 15 F_1_ plants were susceptible indicating recessive nature of resistance. Out of 294 F_2_ plants, 75 plants were resistant with ITs ranging from “;” to “1^++^” while 219 plants showed susceptible reaction (Fig. 1). The F_2_ segregation showed a good fit to theoretically expected ratio of 1 resistant: 3 susceptible plants (χ^2^_(1:3)_ = 0.041, p-value = 0.84) for a single recessive gene. The results were further confirmed in F_2:3_ families. The 284 F_2:3_ families segregated into 1 resistant: 2 segregating: 1 susceptible with χ^2^_(1:2:1)_ = 0.254 (p-value = 0.88). BC_1_ generation also showed expected segregation of IR:1S plants (Table 3).

**Table 3:** Segregation of leaf rust resistance at seedling stage in F_2_, BC_1_F_1_ and F_2:3_ populations against pathotype 77-5 at temperatures range of 20–28 °C.

Generation	Total progeny scored	Number of seedlings/families			Expected ratio	χ^2^_(calc)_	*p* -value
		Resistant	Segregating	Susceptible			
F_2_	294	75	–	219	1R:3S	0.041	0.84
BC_1_F_1_	245	115	–	130	1R:1S	0.918	0.34
F_2:3_	284	70	146	68	1HR:2Seg:1HS	0.254	0.88

### Mapping of leaf rust resistance

**Figure 2:**
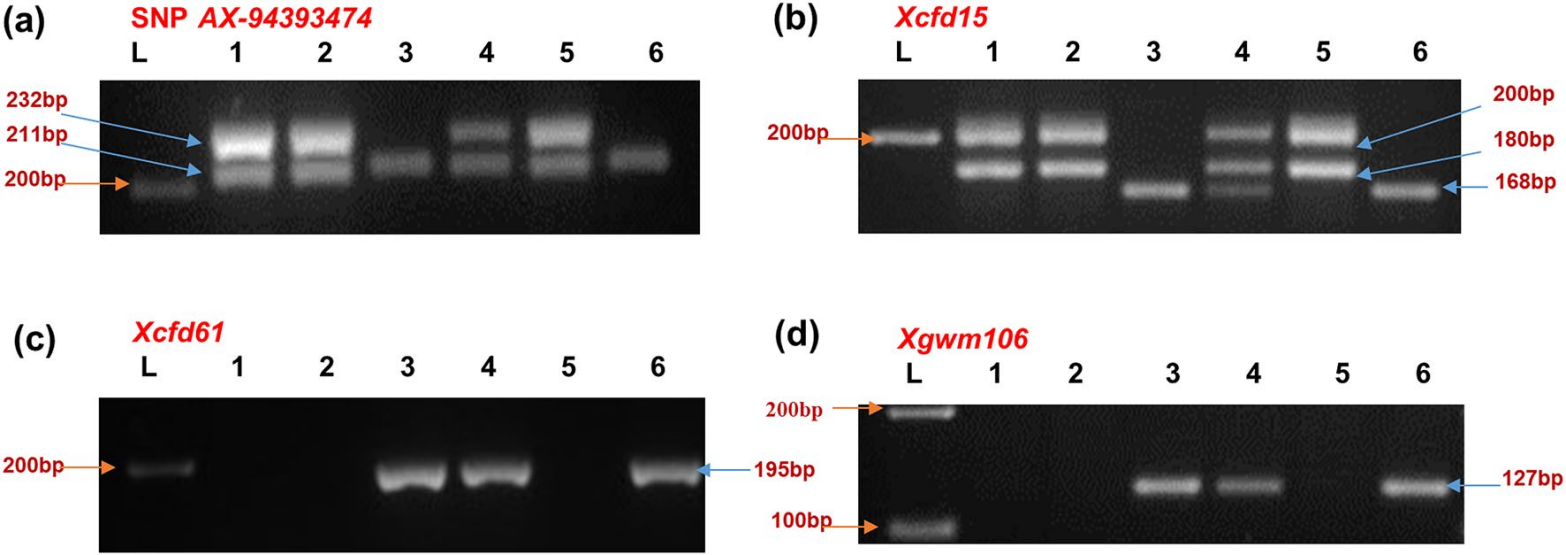
Bulk Segregant Analysis of leaf rust resistance in F_2:3_ population of TSD276-2/AL cross. Lanes: (L) 100 bp ladder, (1) Donor parent *T.spelta* 276, (2) Resistant parent TSD276-2, (3) Susceptible parent AL, (4) F_1_ (TSD276-2/AL), (5) Resistant Bulk, and (6) Susceptible Bulk.

**Figure 3:**
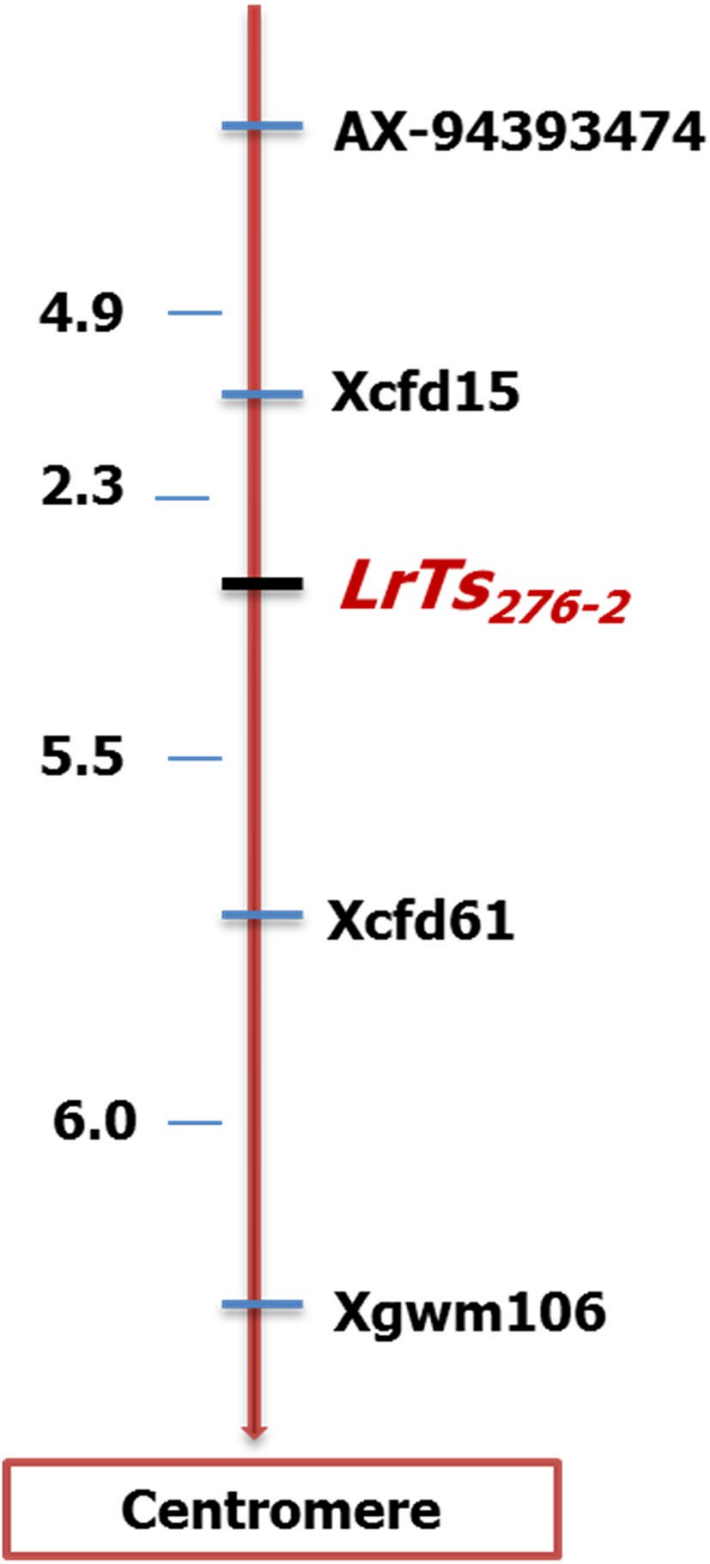
Linkage map of 1DS chromosome in our study based on 136 homozygous F_2:3_ lines of TSD276-2/AL cross.

Genotyping data points for 35,143 SNP markers were obtained, which were filtered in a sequential manner. SNPs lacking any chromosome ID and position were removed. Moreover, SNPs showing heterozygous alleles are also filtered out. Further filtering resulted into 2414 SNPs showing polymorphism between parent viz*.* TSD276-2 and AL. Of these, only 20 SNPs were polymorphic between resistant and susceptible bulks. The 20 polymorphic SNPs were distributed over 10 chromosomes but five SNPs were observed in the short arm of chromosome 1D indicating putative linkage of these SNPs with leaf rust resistance gene in TSD276-2. The five SNPs on chromosome arm 1DS carried the identical alleles in *T. spelta* 276 and TSD276-2 (Table 4) and were converted into SNP-based PCR markers (Table 5). Among SSR markers on chromosome 1D, twelve were polymorphic between parents TSD276-2 and Agra Local. A combined BSA analysis using five SNP-based PCR markers and twelve SSR markers identified one SNP-based PCR marker (*AX-94393474*) and three SSR markers (*Xcfd15, Xcfd61* and *Xgwm106*) as polymorphic between resistant and susceptible bulks (Fig. 2). For construction of linkage map, 136 F_2:3_ families comprising 68 homozygous resistant and 68 homozygous susceptible families were genotyped. Linkage map of leaf rust resistance gene in TSD276-2 was constructed with three SSR and one SNP-based PCR marker covering genetic distance of 18.7 cM on short arm of chromosome 1D (Fig. 3). The leaf rust resistance gene in TSD276-2, hereafter referred as *LrTs*_*276-2*_ is flanked by SSR markers *Xcfd15* and *Xcfd61* spanning 7.8 cM interval on map. SSR marker *Xcfd15* mapped closest to the gene at 2.3 cM. The SNP based PCR marker *AX-94393474* mapped 7.2 cM distal to the gene *LrTs*_*276-2*_. *Xcfd61* mapped 5.5 cM proximal to rust resistance gene. The order of SSR and SNP based marker in the linkage map is consistent with the consensus map of Somers et al. 2004 as well as with CS-IWGSC RefSeq v1 (Table 5).

**Table 4:** AXIOM array SNP genotyping data showing polymorphic SNPs between parents and bulks on 1DS-chromosome.

SNP Probeset_Id	*T. spelta* 276	TSD276-2	AL	RB	SB	IWGSC v1.0 position (bp)
AX-95241170	TT	TT	CC	TT	CC	3,965,001
AX-94393474	CC	CC	AA	CC	AA	3,967,540
AX-94772107	GG	GG	TT	GG	TT	3,969,410
AX-94818846	CC	CC	TT	CC	TT	8,727,512
AX-94570332	–	–	TT	–	TT	21,830,064

**Table 5:** SNP based primers and SSR primers on 1DS-chromosome used in the study.

Markers	Marker type	Designed SNP primer sequence (F&R)	CS-IWGSC RefSeq v1.0 genomic position
*AX-95241170*	SNP based PCR marker	F 5′ AGAATGAGGATGGCAGCGAT 3′ R 5′ CACCACAAATTCACAGGCCA 3′	3,965,001 bp
*AX-94393474*	SNP based PCR marker	F 5′ GAGAGAGATCGATATGTTCTGGAC 3′ R 5′ GGCAGCAAACAGAACCTTCA 3′	3,967,540 bp
*AX-94772107*	SNP based PCR marker	F 5′ GCGTTCGCATGGCGATG 3′ R 5′ ACACCAGTAGCAACCCGTTACCAG 3′	3,969,410 bp
*AX-94818846*	SNP based PCR marker	F 5′ GGTTGCAGAACTTCCTACCG 3′ R 5′ TGCCAGAAGTTGTGCTTTATTGA 3′	8,727,512 bp
*AX-94570332*	SNP based PCR marker	F 5′ GCACAAACAGGCTAACAAAACCTTTA 3′ R 5′ GGGCCCTATTTAGGAGATGTGAC 3′	21,830,064 bp
*Xcfd15*	SSR	F 5′ CTCCCGTATTGAGCAGGAAG 3′ R 5′ GGCAGGTGTGGTGATGATCT 3′	9054 kb
*Xcfd61*	SSR	F 5′ ATTCAAATGCAACGCAAACA 3′ R 5′ GTTAGCCAAGGACCCCTTTC 3′	15,414 kb
*Xgwm106*	SSR	F 5′ CTGTTCTTGCGTGGCATTAA 3′ R 3′ AATAAGGACACAATTGGGATGG 3′	18,188 kb

The SSR marker *Xcfd15,* closest to the resistance gene *LrTs*_*276-2*_ behaved as a codominant marker amplifying alleles of 180 and 200 bp in TSD276-2 and only one allele i.e. 168 bp in AL (Table 6, Supplementary Fig. S1 online). The 200 bp allele of SSR marker *Xcfd15* was found to be linked with the leaf rust resistance in TSD276-2. *T. spelta* 276, the original source of resistance gene *LrTs*_*276-2*_ also amplified alleles identical to TSD276-2. The markers *Xcfd61* and *Xgwm106* were linked with leaf rust resistance gene in repulsion phase and behaved as dominant markers amplifying alleles of 195 bp and 127 bp in susceptible parent AL, respectively (Fig. 2). Both *Xcfd61* and *Xgwm106* produced null allele in TSD276-2 and *T. spelta* 276. The SNP-based PCR marker *AX-94393474* was linked with resistance gene in coupling phase and amplified alleles of 211 and 232 bp in TSD276-2 and *T. spelta* 276 while only single allele i.e. 211 bp was produced in AL showing the dominant nature of marker (see Supplementary Fig. S1 online). Further, the total number of genes between two flanking markers i.e. *cfd15* and *cfd61* observed in *Triticum aestivum* (covering 6.36 Mb sequence) and *Ae. tauschii* (covering 6.21 Mb sequence) were 141 and 84, respectively. Out of these, 45 genes in *Triticum aestivum* and 27 genes in *Ae. tauschii* have R gene related domain (see Supplementary Table S1 and Supplementary Table S2 online). Among these, 24 genes are common between *Triticum aestivum* and *Ae. tauschii* between two flanking markers (see Supplementary Table S3 online).

**Table 6:** Polymorphic allele size scored between parents and the bulks and used in mapping population screening.

Markers	Alleles amplified (in bp)			Polymorphic allele used in mapping (TSD276-2/AL)
	*T. spelta* 276	TSD276-2	Agra local	
SNP *AX-94393474*	211, 232	211, 232	211	232/–
*Xcfd15*	180, 200	180, 200	168	200/168
*Xcfd61*	–	–	195	–/195
*Xgwm106*	–	–	127	–/127

## Discussion

The leaf rust resistance gene in TSD276-2 showed a wide spectrum of resistance against Indian *P. triticina* pathotypes. Genetic analysis showed a single recessive gene conferring leaf rust resistance in TSD276-2. The resistance gene was mapped on short arm of chromosome 1D and was flanked by SSR markers *Xcfd15* and *Xcfd61*. The resistance gene in TSD276-2 is derived from the spelt wheat accession *T. spelta*276. A large number of rust resistance genes have been transferred into wheat from the species belonging to secondary and tertiary gene pools. These resistance genes often carry some degree of linkage drag and sometimes undesirable genes^17,29,30^. Genetic resources from primary gene pool have the advantage of homologous recombination which can be used to remove the linkage drag. Closely related species of wheat from primary gene pool are rich and diverse source of unique alleles that can be used in wheat improvement^12,31–35^. In the present study, we identified a seedling leaf rust resistance gene tentatively named as *LrTs*_*276-2*_ in spelt wheat derived common wheat line TSD276-2. Till date, only three leaf rust resistance genes from spelt wheat viz., *Lr44*, *Lr65* and *Lr71* have been identified and mapped. While *Lr44* and *Lr71* have been located on chromosome 1B^10,13^, *Lr*65 has been mapped on chromosome 2A^12^. The leaf rust resistance gene in the present study has been mapped on short arm of chromosome 1D indicating that *LrTs*_*276-2*_ is different from the already characterized leaf rust resistance genes from spelt wheat and potentially a novel leaf rust resistance gene. Moreover, *Lr44*, *Lr65* and *Lr71* behaved as dominant genes while the *LrTs*_*276-2*_ in TSD276-2 is recessive in nature, differentiating this gene from other spelt wheat genes characterized so far. *Lr65* has also been reported to be susceptible to Indian pathotype 77-5^19^.

**Figure 4:**
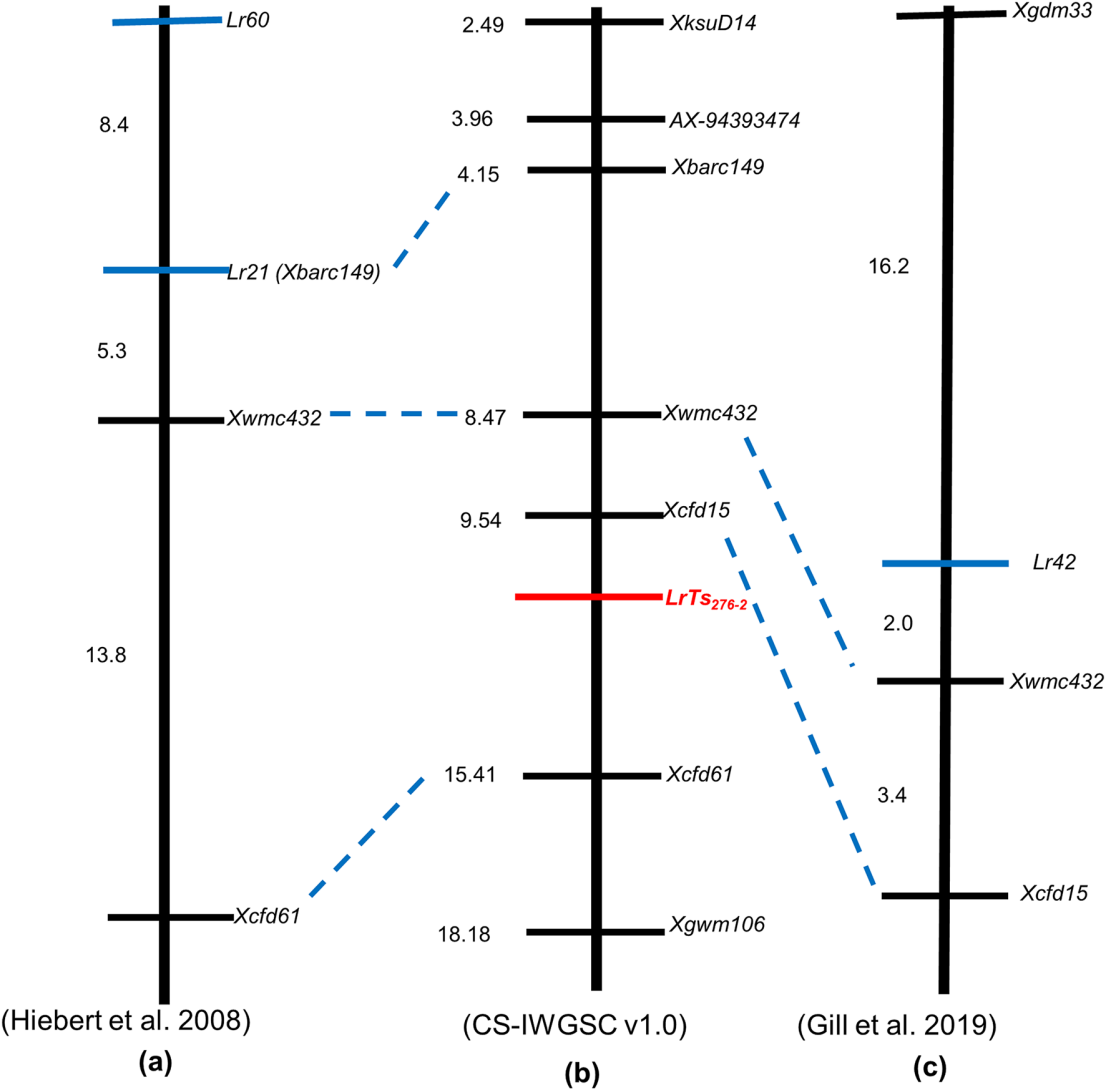
Comparative analysis of *LrTs*_*276*-2_ along with *Lr42, Lr60* and *Lr21.* Map unit is cM in (**a**) and (**c**), Map unit is Mbp in (**b**).

Till date, three leaf rust resistance genes viz., *Lr21*^36,37^, *Lr42*^36,38–40^ and *Lr60*^41^ have been mapped on 1DS chromosome of wheat. Among these genes *Lr21* and *Lr42* have been transferred in common wheat from diploid progenitor species *Aegilops tauschii* (2n = 2x = 14, genome DD)^36,37,40,42^ while *Lr60* is native in bread wheat^41,43^. Although the gene *LrTs*_*276-2*_ mapped by us is from *T. spelta* and is expected to be different, nevertheless it is important to distinguish this gene from other leaf rust resistance genes mapped on chromosome 1DS. This can be done on the basis of differential response to *P. triticina* pathotypes and by comparing the genetic and physical position on the chromosome. The gene *Lr21* is ineffective against several Indian pathotypes of *P. triticina*^44^ whereas in our study both *T. spelta* 276 and TSD276-2 showed high degree of resistance against all the 17 pathotypes used in the study. The response of *Lr60* to Indian pathotypes of *P. triticina* is not available. However, *Lr42* shows resistance to all the *P. triticina* pathotypes in India^44^. Hiebert et al.^41^ analyzed the linkage between *Lr21* and *Lr60* and observed that *Lr60* is about 13.5 cM distal to *Lr21* with the SSR marker *Xbarc149* co-segregating with *Lr21*. *Lr42* has been reported as race-specific partially dominant resistance gene^36^. However, Czembor et al.^45^ mapped *Lr42* on chromosome 3D and observed that *Lr42* behaved as dominant gene. Sun et al.^38^ mapped *Lr42* on the distal end of chromosome arm 1DS and marker *Xwmc432* was found closest to the gene *Lr42* at a distance of 0.8 cM. Liu et al.^39^ reported *Lr42* as recessive gene and mapped it on 1DS chromosome with flanking markers *Xwmc432* and *Xgdm33* spanning a genetic distance of 17 cM. *Xwmc432* was the closest marker 4 cM proximal to *Lr42*. Gill et al.^40^ narrowed down the *Lr42* region to 3.7 cM with marker TC387992 at a distance of 1.7 cM distal to *Lr42* and *Xwmc432* located at 2 cM proximal to *Lr42.* The gene *LrTs*_*276-2*_ mapped by us in TSD276-2 is flanked by the markers *Xcfd15* and *Xcfd61*. The gene *LrTs*_*276-2*_ is 2.3 cM proximal to marker *Xcfd15* while Gill et al.^40^ reported *Lr42* at a distance of 5.4 cM distal to *Xcfd15*. Thus, *Lr42* is located distal to *LrTs*_*276-2*_ on chromosome arm 1DS. A comparison of genetic and physical maps unambiguously shows that the locus represented by *LrTs*_*276-2*_ is different from other rust resistance loci mapped on chromosome 1DS (Fig. 4). Anchoring of markers linked to *Lr21*, *Lr 42*, *Lr60* and *LrTs*_*276-2*_ on Chinese Spring Reference genome indicates that these genes are located on 1DS chromosome in order of *Lr60*-*Lr21*-*Lr42* and *LrTs*_*276-2*_. Further, in-silico studies suggested that the flanking markers are more than 6 Mb apart. The predicted number of genes related to disease resistance with R gene domain (using domain reported by Peng et al.^46^) in the species i.e. *Triticum aestivum* and *Aegilops tauschii* are very high. Hence, it is essential to narrow down the region for prediction of putative candidate gene. The rust reaction, nature of gene and comparative genomics indicates that *LrTs*_*276-2*_ is a novel leaf rust resistance gene that may be useful in resistance breeding programs in wheat.

## Supplementary Information

Supplementary Information 1.

Supplementary Information 2.
