# Supplementary material for: Molecular mapping of a new recessive wheat leaf rust resistance gene originating from Triticum spelta
Source: Sci Rep. 2020 Dec 17;10:22113. doi: 10.1038/s41598-020-78679-3 (PMC7746701; doi:10.1038/s41598-020-78679-3)

Molecular mapping of a new recessive wheat leaf rust resistance gene originating from *Triticum spelta*

Vishal Dinkar, S.K. Jha, Niharika Mallick, M. Niranjana, Priyanka Agarwal, J.B. Sharma and Vinod*

Division of Genetics, ICAR- Indian Agricultural Research Institute, New Delhi-110012, India

1. Vishal Dinkar <vishaldinkar1991@gmail.com>,

   Division of Genetics, ICAR-Indian Agricultural Research Institute

2. S K Jha <jhashail78@gmail.com>

   Division of Genetics, ICAR-Indian Agricultural Research Institute

3. Niharika Mallick <niharikamallick@gmail.com>

   Division of Genetics, ICAR-Indian Agricultural Research Institute

4. M Niranjana <mniranjana2010@gmail.com>

   Division of Genetics, ICAR-Indian Agricultural Research Institute

5. Priyanka Agarwal <priyankaagarwal11@gmail.com>

   Division of Genetics, ICAR-Indian Agricultural Research Institute

6. Jai Sharma <jbsiari@gmail.com>

   Division of Genetics, ICAR-Indian Agricultural Research Institute

7. Vinod (corresponding author) <vinod.genetics@gmail.com>

   Division of Genetics, ICAR-Indian Agricultural Research Institute

Supplementary Information 2: Full-length gels of Supplementary figure S1 (a-h) online. Red squares are marking cropped parts of gels presented in the Supplementary figure S1.

Supplementary figure S1- a and b:

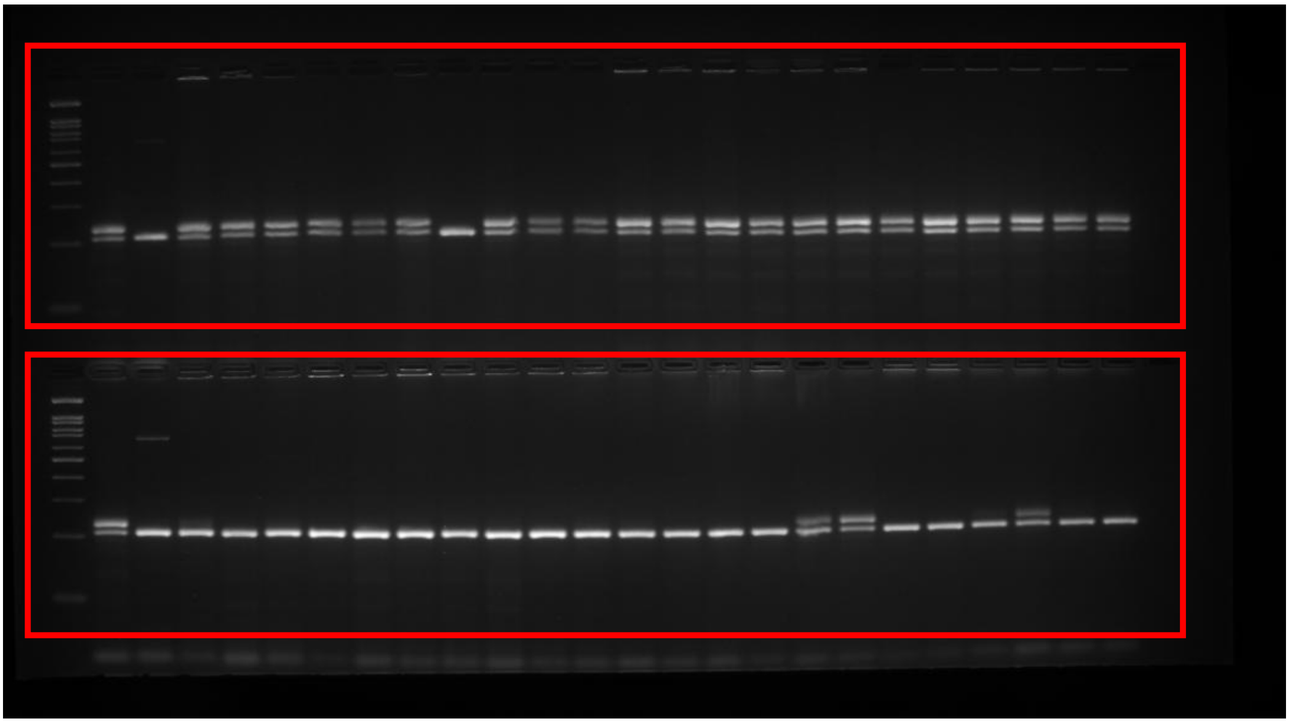

Supplementary figure S1- c and d:

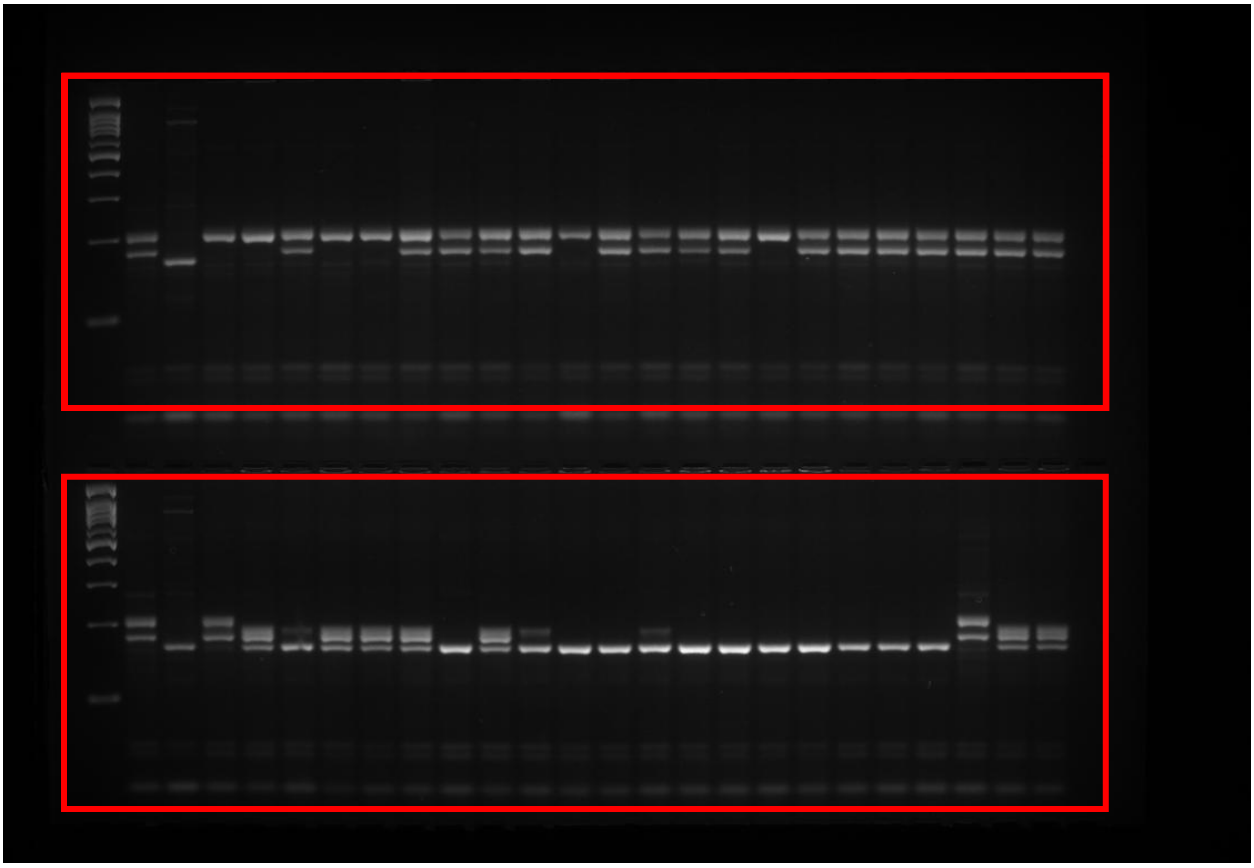

Supplementary figure S1- e:

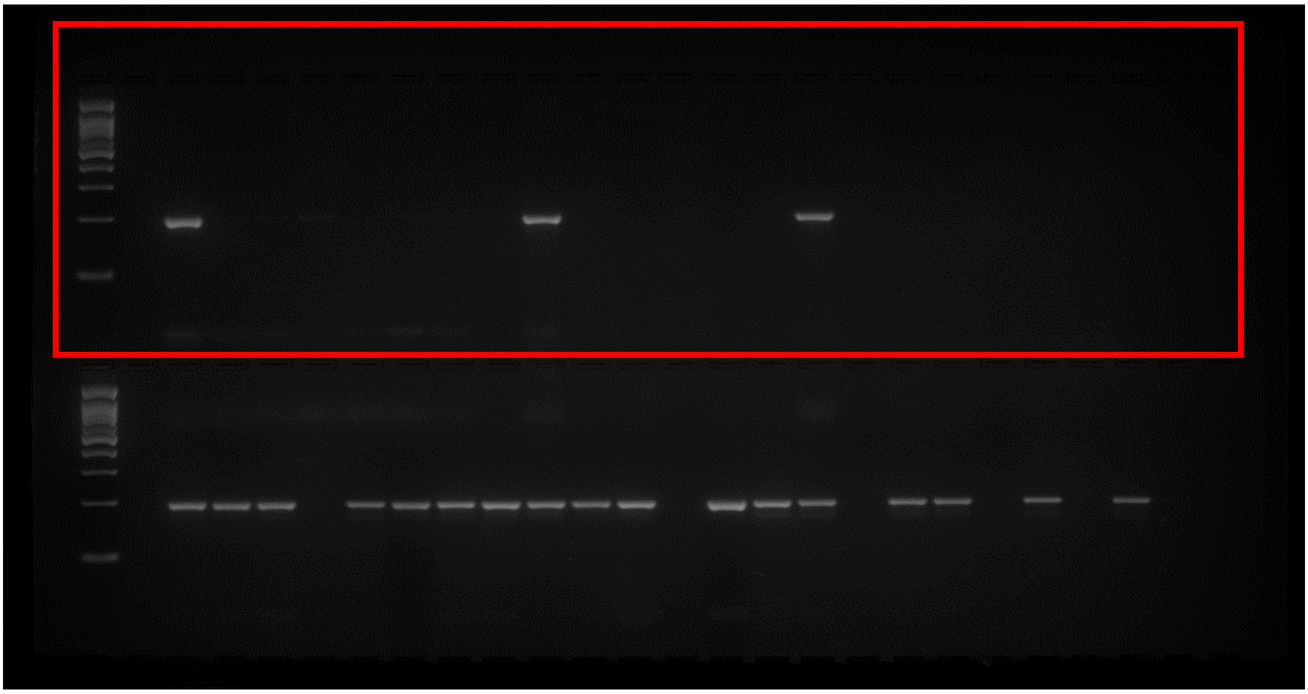

Supplementary figure S1- f:

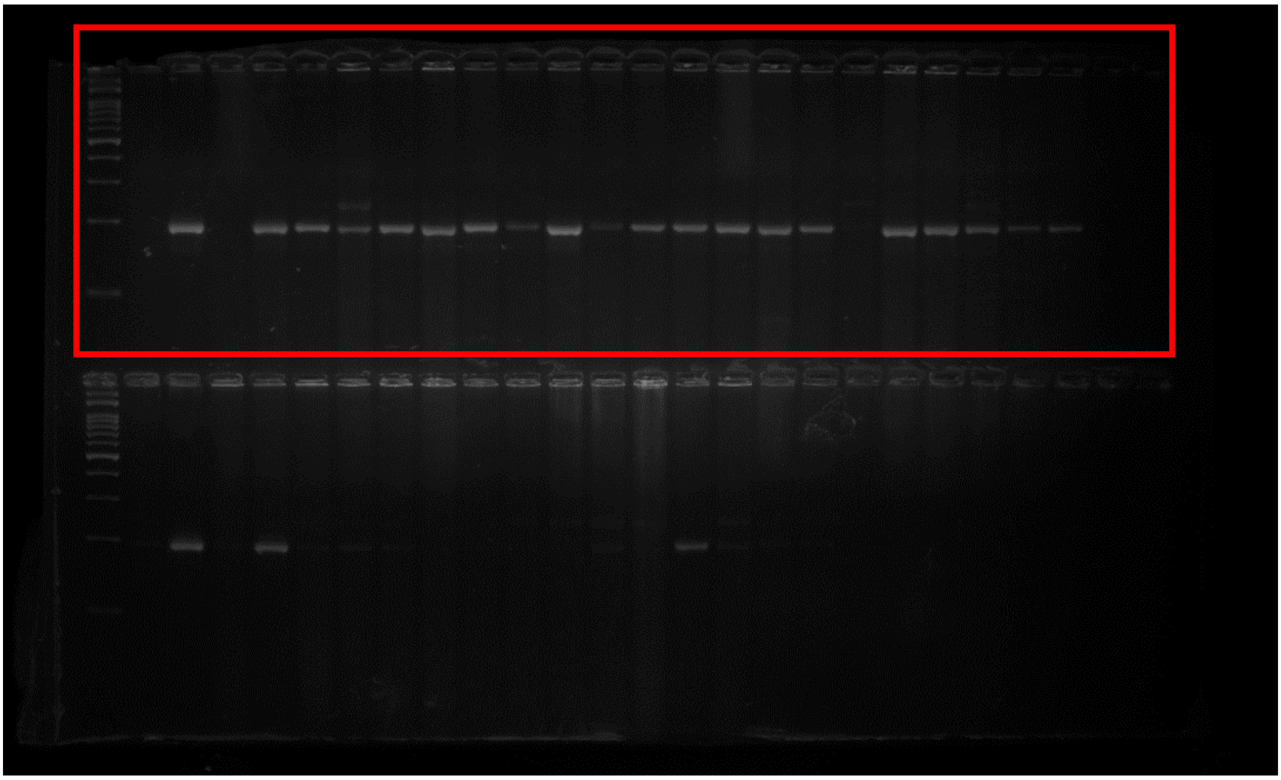

Supplementary figure S1- g:

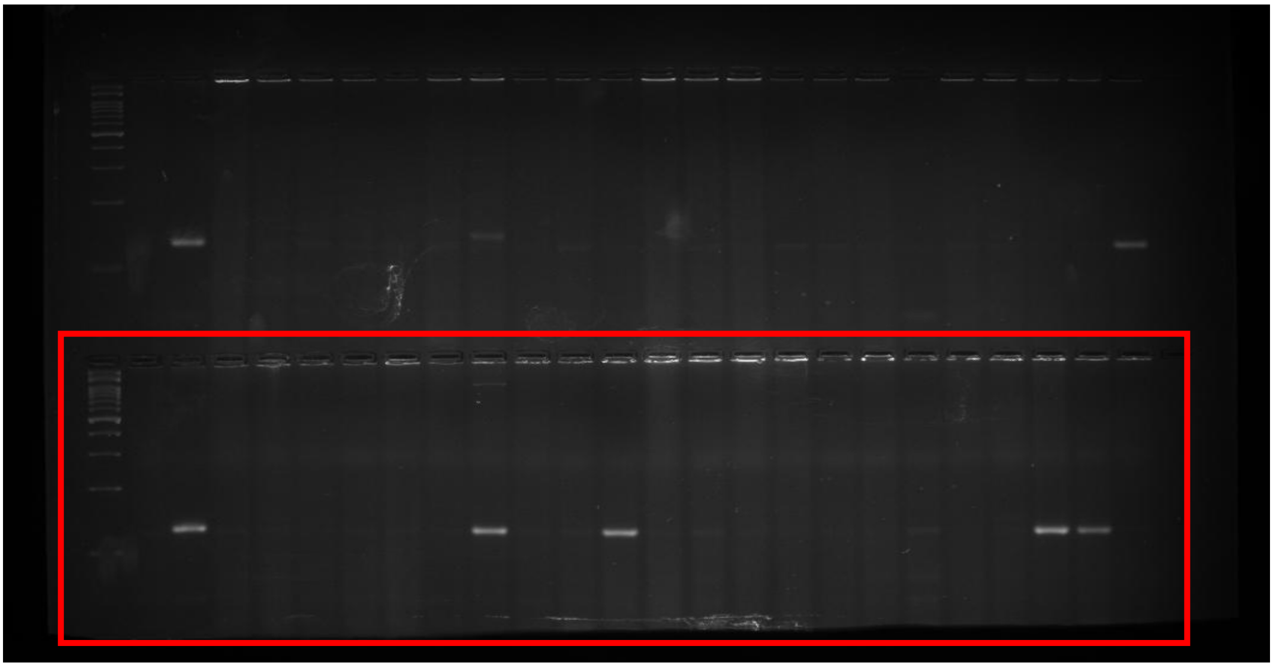

Supplementary figure S1- h:

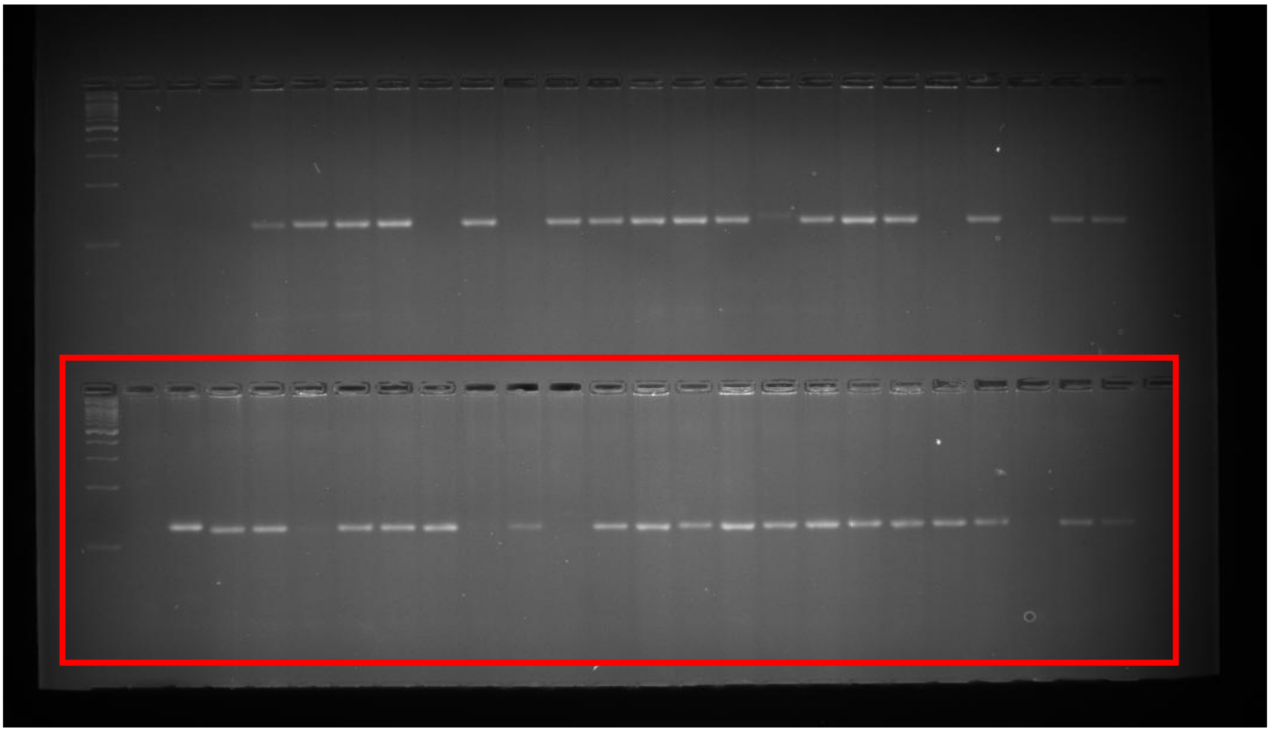

Supplementary Information 3: Full-length gels of Figure 2 (a-d). Red squares are marking cropped parts of gels presented in the manuscript.

Figure 2 (a):

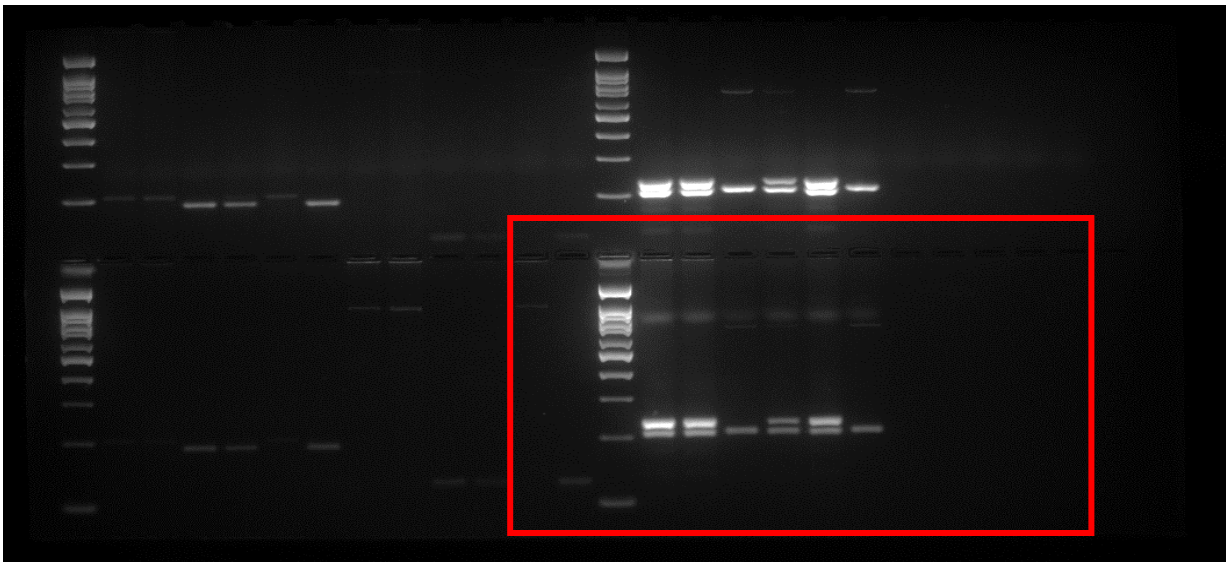

Figure 2 (b):

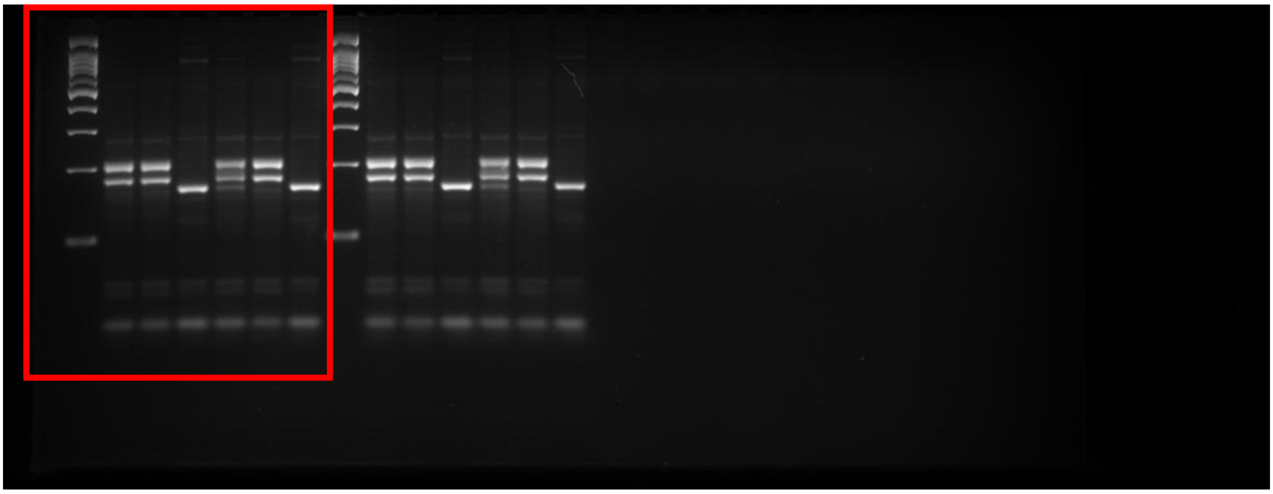

Figure 2 (c) and (d):

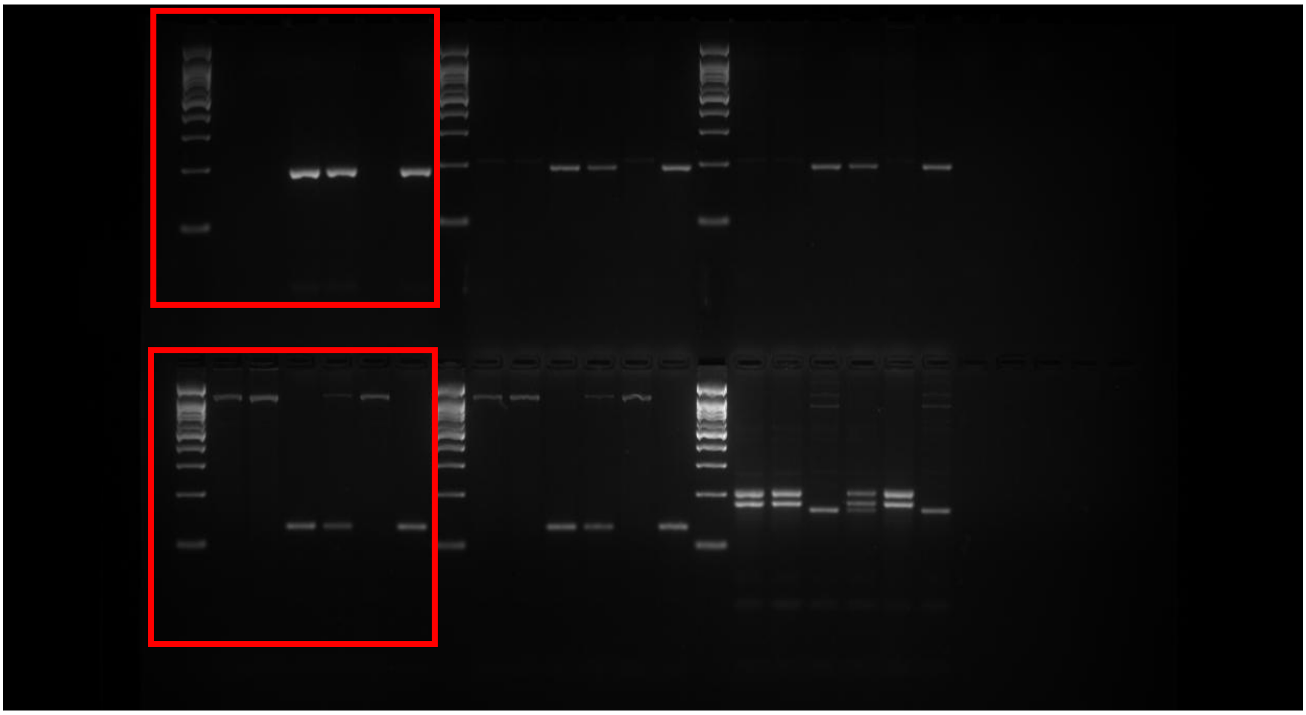

Supplement: Supplementary file 1 — Supplementary Information 1. [file 41598_2020_78679_MOESM1_ESM.pdf]
