# Supplementary material for: Molecular mapping of a new recessive wheat leaf rust resistance gene originating from Triticum spelta
Source: Sci Rep. 2020 Dec 17;10:22113. doi: 10.1038/s41598-020-78679-3 (PMC7746701; doi:10.1038/s41598-020-78679-3)
Supplement: Supplementary file 2 — Supplementary Information 2. [file 41598_2020_78679_MOESM2_ESM.pdf]

Supplementary Information

Molecular mapping of a new recessive wheat leaf rust resistance gene originating from *Triticum spelta*

Vishal Dinkar, S.K. Jha, Niharika Mallick, M. Niranjana, Priyanka Agarwal, J.B. Sharma and Vinod*

Division of Genetics, ICAR- Indian Agricultural Research Institute, New Delhi-110012, India

*Corresponding Author: Vinod; E-mail: vinod.genetics@gmail.com

Supplementary Figure S1. Screening of $F_{2:3}$ population. (a&b) SNP *AX-94393474* based PCR marker scored as dominant marker; (c&d) SSR marker *Xcfd15* scored as codominant marker, (e&f) SSR marker *Xcfd61* scored as dominant marker, (g&h) SSR marker *Xgwm106* scored as dominant marker. *Abbrev.*: L (100bp ladder), RP (Resistant parent, TSD276-2), SP (Susceptible parent, Agra Local), Yellow arrows showing resistant and susceptible parent alleles, Re (Recombinant individual).

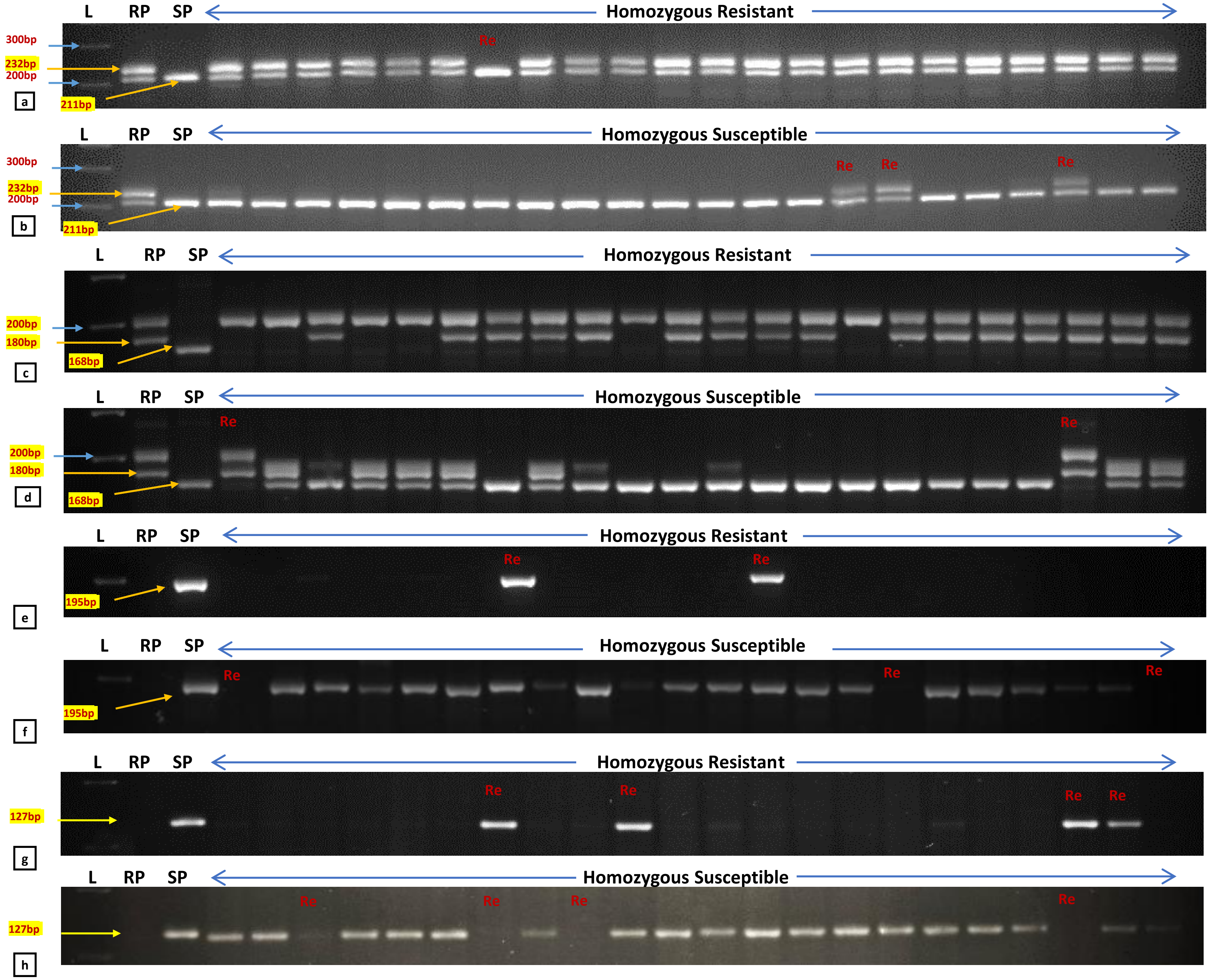

Supplementary Table S1. Candidate gene(s) identified related to disease stress in the marker interval region of *Xcfd15* and *Xcfd61*

S.No.	Gene stable ID	Interpro Description	UniProtKB/ TrEMBL ID	Gene start (bp)	Gene end (bp)
1	*TraesCS1D02G021200*	NB-ARC, P-loop containing nucleoside triphosphate hydrolase	A0A341PBA9	9086119	9091764
2	*TraesCS1D02G021700*	Homeobox-like domain superfamily	W5ALN8	9283505	9288824
3	*TraesCS1D02G022500*	NB-ARC, P-loop containing nucleoside triphosphate hydrolase, Leucine-rich repeat domain superfamily	A0A1D5T4I5	9575753	9593333
4	*TraesCS1D02G023000*	Leucine-rich repeat domain superfamily	A0A341PBC9	9774410	9779817
5	*TraesCS1D02G023100*	Leucine-rich repeat domain superfamily	A0A1D5T214	9799492	9811625
6	*TraesCS1D02G023700*	P-loop containing nucleoside triphosphate hydrolase	A0A341PF47	10024701	10027846
7	*TraesCS1D02G024400*	ABC transporter-like,AAA+ ATPase domain, P-loop containing nucleoside triphosphate hydrolase, ABC transporter-like	W5ANB2	10257122	10268568
8	*TraesCS1D02G024500*	Leucine-rich repeat domain superfamily	W5AL46	10292749	10295636
9	*TraesCS1D02G025200*	NB-ARC, P-loop containing nucleoside triphosphate hydrolase	A0A1D5T1Z3	10408161	10408847
10	*TraesCS1D02G025500*	Ankyrin repeat-containing domain	A0A341PF57	10515553	10517008
11	*TraesCS1D02G025600*	P-loop containing nucleoside triphosphate hydrolase	A0A341PDC7	10548189	10549302
12	*TraesCS1D02G025700*	Ankyrin repeat	A0A1D5T498	10599221	10603768
13	*TraesCS1D02G026000*	NB-ARC, P-loop containing nucleoside triphosphate hydrolase, Leucine-rich repeat domain superfamily		10661025	10664946
14	*TraesCS1D02G026100*	Zinc finger, RING/FYVE/PHD-type	A0A1D6RHH5	10666322	10671961
15	*TraesCS1D02G026200*	Protein kinase domain,Protein kinase-like domain superfamily	A0A1D5T1R3	10715309	10722269
16	*TraesCS1D02G026800*	P-loop containing nucleoside triphosphate hydrolase	A0A341PMZ5	10808283	10810373
17	*TraesCS1D02G028200*	NB-ARC, P-loop containing nucleoside triphosphate hydrolase, Leucine-rich repeat domain superfamily	A0A1D6DF23	11175841	11182532
18	*TraesCS1D02G028600*	NB-ARC, P-loop containing nucleoside triphosphate hydrolase, Leucine-rich repeat domain superfamily	A0A1D5T2S3	11272449	11278362
19	*TraesCS1D02G028700*	Leucine-rich repeat domain superfamily	A0A341PN12	11287245	11292876
20	*TraesCS1D02G028736*	Leucine-rich repeat domain superfamily		11319796	11321348
21	*TraesCS1D02G028800*	NB-ARC, P-loop containing nucleoside triphosphate hydrolase, Leucine-rich repeat domain superfamily	W5ALK5	11397758	11401635
22	*TraesCS1D02G029000*	NB-ARC, P-loop containing nucleoside triphosphate hydrolase, Leucine-rich repeat domain superfamily	A0A1D5T240	11408761	11415088
23	*TraesCS1D02G029100*	NB-ARC, P-loop containing nucleoside triphosphate hydrolase, Leucine-rich repeat domain superfamily	S5DMA6	11451423	11459353
24	*TraesCS1D02G029200*	NB-ARC, P-loop containing nucleoside triphosphate hydrolase, Leucine-rich repeat domain superfamily	A0A1D6RGU0	11493627	11499140

25	*TraesCS1D02G029400*	Armadillo-like helical, Armadillo-type fold	W5AML7	11543975	11550887
26	*TraesCS1D02G029900*	NB-ARC, P-loop containing nucleoside triphosphate hydrolase, Leucine-rich repeat domain superfamily	A0A1D6RGF3	11654673	11662254
27	*TraesCS1D02G030000*	Protein kinase domain,Protein kinase-like domain superfamily	A0A341PN32	11664577	11668738
28	*TraesCS1D02G030041*	NB-ARC, P-loop containing nucleoside triphosphate hydrolase, Leucine-rich repeat domain superfamily	A0A1D5T1F2	11679306	11683061
29	*TraesCS1D02G030300*	Zinc finger, RING/FYVE/PHD-type	A0A1D5T1E8	11780392	11783116
30	*TraesCS1D02G030700*	Transcription factor GRAS	A0A341PDI4	11973553	11977690
31	*TraesCS1D02G030800*	Transcription factor GRAS	A0A341PE08	12029858	12031963
32	*TraesCS1D02G030900*	Transcription factor GRAS	A0A341PBL6	12054846	12056987
33	*TraesCS1D02G031000*	Transcription factor GRAS	A0A341PN41	12141576	12143753
34	*TraesCS1D02G031200*	Transcription factor GRAS	A0A1D5T212	12254358	12256547
35	*TraesCS1D02G031300*	Protein kinase-like domain superfamily, Serine-threonine/tyrosine-protein kinase, catalytic domain	A0A1D5T2U3	12299616	12303935
36	*TraesCS1D02G031400*	P-loop containing nucleoside triphosphate hydrolase	A0A1D5T2U5	12310956	12314930
37	*TraesCS1D02G031800*	P-loop containing nucleoside triphosphate hydrolase	A0A341PE17	12335159	12337241
38	*TraesCS1D02G032200*	NB-ARC, P-loop containing nucleoside triphosphate hydrolase, Leucine-rich repeat domain superfamily	A0A1D6RH97	12532041	12536914
39	*TraesCS1D02G032600*	Protein kinase-like domain superfamily	W5AK16	13028775	13032480
40	*TraesCS1D02G033200*	Serine-threonine/tyrosine-protein kinase, catalytic domain, Protein kinase-like domain superfamily, Leucine-rich repeat domain superfamily	A0A1D5T4I0	13311429	13316106
41	*TraesCS1D02G033300*	Serine-threonine/tyrosine-protein kinase, catalytic domain, Protein kinase-like domain superfamily, Leucine-rich repeat domain superfamily,Leucine-rich repeat domain superfamily	A0A341PDL6	13320662	13325391
42	*TraesCS1D02G033400*	Serine-threonine/tyrosine-protein kinase, catalytic domain, Protein kinase domain superfamily	A0A341PE35	13362256	13368718
43	*TraesCS1D02G033500*	Serine-threonine/tyrosine-protein kinase, catalytic domain, Protein kinase domain superfamily	A0A1D5T2S2	13503088	13508137
44	*TraesCS1D02G034100*	Leucine-rich repeat domain superfamily,cysteine-containing subtype	W5AMA1	14476778	14479949
45	*TraesCS1D02G034705*	Leucine-rich repeat domain superfamily		15293910	15296609

Supplementary Table S2. Candidate gene(s) identified related to disease stress in the marker interval region of *Xcfd15* and *Xcfd61 in Aegilops tauschii*

S.No.	Gene stable ID	Interpro Description	UniProtKB/TrEMBL ID	Gene start (bp)	Gene end (bp)
1	*AET1Gv20046700*	NB-ARC	M8C4R7	10408524	10413615
2	*AET1Gv20049100*	NB-ARC	M8BW38	10872508	10888688
3	*AET1Gv20050200*	P-loop containing nucleoside triphosphate hydrolase		11049923	11051317
4	*AET1Gv20050600*	Leucine-rich repeat, typical subtype	M8AZJ4	11117242	11132638
5	*AET1Gv20053000*	ABC transporter-like	M8C4Y6	11639164	11688106
6	*AET1Gv20053100*	Protein kinase domain	M8C5I5	11714612	11718925
7	*AET1Gv20054300*	NB-ARC		11789227	11793145
8	*AET1Gv20054700*	NB-ARC	M8C7F9	11878051	12013597
9	*AET1Gv20054900*	Harbinger transposase-derived nuclease domain		11980435	11980889
10	*AET1Gv20055200*	Zinc finger, RING-type	M8BW71	12015944	12021558
11	*AET1Gv20055400*	Protein kinase domain	M8BBL6	12027742	12036524
12	*AET1Gv20056300*	Ankyrin repeat	M8B2T0	12211251	12217848
13	*AET1Gv20057200*	P-loop containing nucleoside triphosphate hydrolase	M8BW73	12312037	12317361
14	*AET1Gv20059000*	Leucine-rich repeat domain superfamily	M8ARP5	12508989	12611130
15	*AET1Gv20060200*	NB-ARC	M8B7H9	12702219	12711934
16	*AET1Gv20061800*	NB-ARC	M8C837	13008389	13011980
17	*AET1Gv20061900*	Zinc finger, RING-type	M8CIT0	13013735	13015193
18	*AET1Gv20063000*	Transcription factor GRAS	M8BGZ5	13226724	13230953
19	*AET1Gv20063300*	P-loop containing nucleoside triphosphate hydrolase	R7WG24	13265513	13328057
20	*AET1Gv20064200*	P-loop containing nucleoside triphosphate hydrolase	R7WB76	13344111	13345393
21	*AET1Gv20064800*	NB-ARC	M8AWJ5	13512008	13524327
22	*AET1Gv20065700*	Protein kinase domain	M8BND7	13987571	13991504
23	*AET1Gv20066500*	Protein kinase domain	M8ANN0	14259160	14298192
24	*AET1Gv20066800*	Protein kinase domain	N1QZA5	14417318	14422467
25	*AET1Gv20067400*	Protein kinase-like domain superfamily		15206334	15207025
26	*AET1Gv20067800*	Leucine-rich repeat, cysteine-containing subtype	M8BC59	15454609	15457781
27	*AET1Gv20070800*	Homeobox domain	M8C863	16593488	16594114

Supplementary Table S3. List of twenty-four common candidate gene(s) between *Aegilops tauschii* and *Triticum aestivum*

	Aegilops tauschii			*Triticum aestivum*		
S.No.	**Gene stable ID**	**Gene start (bp)**	**Gene end (bp)**	**Gene stable ID**	**Gene start (bp)**	**Gene end (bp)**
1	AET1Gv20046700	10408524	10413615	*TraesCS1D02G021200*	9086119	9091764
2	AET1Gv20049100	10872508	10888688	*TraesCS1D02G022500*	9575753	9593333
3	AET1Gv20050200	11049923	11051317	*TraesCS1A02G023200*	11530146	11531462
4	*AET1Gv20050600*	11117242	11132638	*TraesCS1D02G023100*	9799492	9811625
5	*AET1Gv20053000*	11639164	11688106	*TraesCS1D02G024400*	10257122	10268568
6	*AET1Gv20053100*	11714612	11718925	*TraesCS1A02G024400*	12002939	12006839
7	*AET1Gv20054700*	11878051	12013597	*TraesCS1D02G026000*	10661025	10664946
8	*AET1Gv20055200*	12015944	12021558	*TraesCS1D02G026100*	10666322	10671961
9	*AET1Gv20055400*	12027742	12036524	*TraesCS1D02G026200*	10715309	10722269
10	*AET1Gv20056300*	12211251	12217848	*TraesCS1D02G025700*	10599221	10603768
11	*AET1Gv20057200*	12312037	12317361	*TraesCSU02G002600*	2567992	2573565
12	*AET1Gv20059000*	12508989	12611130	*TraesCS1A02G028500*	13346605	13352572
13	*AET1Gv20060200*	12702219	12711934	*TraesCS1D02G029200*	11493627	11499140
14	*AET1Gv20061800*	13008389	13011980	*TraesCS1D02G025200*	10408161	10408847
15	*AET1Gv20061900*	13013735	13015193	*TraesCS1D02G030300*	11780392	11783116
16	*AET1Gv20063000*	13226724	13230953	*TraesCS1D02G030900*	12054846	12056987
17	*AET1Gv20063300*	13265513	13328057	*TraesCS1D02G031400*	12310956	12314930
18	*AET1Gv20064200*	13344111	13345393	*TraesCS1D02G031800*	12335159	12337241
19	*AET1Gv20064800*	13512008	13524327	*TraesCS1D02G032200*	12532041	12536914
20	*AET1Gv20065700*	13987571	13991504	*TraesCS1D02G032600*	13028775	13032480
21	*AET1Gv20066500*	14259160	14298192	*TraesCS1D02G033200*	13311429	13316106
22	*AET1Gv20066800*	14417318	14422467	*TraesCS1D02G033500*	13503088	13508137
23	*AET1Gv20067400*	15206334	15207025	*TraesCS3A02G032900*	18851466	18854370
24	*AET1Gv20067800*	15454609	15457781	*TraesCS1D02G034100*	14476778	14479949
